# Bioactive Coatings Based on Hydroxyapatite, Kanamycin, and Growth Factor for Biofilm Modulation

**DOI:** 10.3390/antibiotics10020160

**Published:** 2021-02-05

**Authors:** Oana Gherasim, Alexandru Mihai Grumezescu, Valentina Grumezescu, Irina Negut, Marius Florin Dumitrescu, Miruna Silvia Stan, Ionela Cristina Nica, Alina Maria Holban, Gabriel Socol, Ecaterina Andronescu

**Affiliations:** 1 Department of Science and Engineering of Oxide Materials and Nanomaterials, Faculty of Applied Chemistry and Materials Science, Politehnica University of Bucharest, 011061 Bucharest, Romania; oana.gherasim@inflpr.ro (O.G.); grumezescu@yahoo.com (A.M.G.); marius.dumitrescu2213@gmail.com (M.F.D.); miruna_stan@yahoo.com (M.S.S.); ecaterina.andronescu@upb.ro (E.A.); 2 Lasers Department, National Institute for Lasers, Plasma and Radiation Physics, 077125 Magurele, Romania; negut.irina@inflpr.ro (I.N.); gabriel.socol@inflpr.ro (G.S.); 3 Research Institute of the University of Bucharest–ICUB, University of Bucharest, 050657 Bucharest, Romania; cristinai.nica@gmail.com (I.C.N.); alina_m_h@yahoo.com (A.M.H.); 4 Department of Biochemistry and Molecular Biology, Faculty of Biology, University of Bucharest, 050095 Bucharest, Romania; 5 Department of Microbiology and Immunology, Faculty of Biology, University of Bucharest, 077206 Bucharest, Romania

**Keywords:** metallic implants, antimicrobial coatings, bioactive materials, improved osseointegration, multifunctional device

## Abstract

The occurrence of opportunistic local infections and improper integration of metallic implants results in severe health conditions. Protective and tunable coatings represent an attractive and challenging selection for improving the metallic devices’ biofunctional performances to restore or replace bone tissue. Composite materials based on hydroxyapatite (HAp), Kanamycin (KAN), and fibroblast growth factor 2 (FGF2) are herein proposed as multifunctional coatings for hard tissue implants. The superior cytocompatibility of the obtained composite coatings was evidenced by performing proliferation and morphological assays on osteoblast cell cultures. The addition of FGF2 proved beneficial concerning the metabolic activity, adhesion, and spreading of cells. The KAN-embedded coatings exhibited significant inhibitory effects against bacterial biofilm development for at least two days, the results being superior in the case of Gram-positive pathogens. HAp-based coatings embedded with KAN and FGF2 protein are proposed as multifunctional materials with superior osseointegration potential and the ability to reduce device-associated infections.

## 1. Introduction

Titanium (Ti) and its alloys represent suitable candidates for the fabrication of implantable devices intended to restore (plates, screws, nails, and wires) [1,2,3,4] and replace (bone implants, joint prostheses, and dental abutments) [5,6,7] severely damaged hard tissues or bone losses. In addition to excellent intrinsic biocompatibility, such materials possess tunable composition (alloying process guided by the final application) and versatile structure (compact or porous microstructure depending on the final product, such as fixation elements, cortical bone and trabecular bone replacements, respectively), which are beneficial for the development of various orthopedic and orthodontic implantable devices. Superior biomechanical properties (increased fatigue strength, fracture toughness, rigidity, stiffness, and wear resistance), general stability, favorable thermochemical behavior, and good corrosion resistance (surface protection ascribed to the native oxide layer) recommend Ti-based materials for manufacturing implants used to partially or completely replace the injured bone tissue [8,9,10,11,12]. However, clinical limitations of Ti and Ti alloys for orthopedic and orthodontic uses rely on their intrinsic inertness and poor biological activity [13,14]. Modern healthcare practice and research provide attractive approaches towards overcoming the bioinertness and limited bioactivity of classical metallic biomaterials. Surface modification and surface coating are versatile strategies to mediate the osseointegration of Ti-based implantable devices through osteoconductivity, osteoinductivity, and osteogenesis potential [15,16].

The osseointegration process of metallic implants, centered on forming a direct interface between the implanted device and the host bone tissue, is important for the stabilization and long-term biomechanical and functional performance of most implants. This complex process consists in (i) early-stage osseointegration (up to one month)—an intimate mechanical-based connection established between the implant and surrounding tissues, leading to initial interactions with resident cells and (ii) late-stage osseointegration (up to six months)—a dynamic molecular and cellular phase resulting in regional bone healing, new bone formation and bone remodeling [17,18].

To support proper osseointegration, the implantation protocol should be considered as soon as possible after severe bone tissue damage has occurred, regardless of the type and nature of the selected replacement. Alternative techniques are available to locally induce a favorable implantation environment, such as cementation [19,20,21] and stem cells therapy [22,23,24] in the case of orthopedic implants, alveolar ridge augmentation [25,26,27,28] and sinus augmentation [29,30,31,32] in the case of orthodontic implants.

Many efforts have been made to enhance metallic implants’ osteogenic activity, such as alloying or composite development, surface texturing or modification, and surface functionalization or coating [33,34]. A common route to boost the integration of metallic implants and to simultaneously overcome their intrinsic bioinertness is to modify their surface with bioactive layers having osteoconductive or/and osteoinductive capabilities [35,36]. 

Surface modification of metallic implants by calcium phosphate capping is an appropriate choice to enhance implant integration and improve biofunctional performance [37,38]. In particular, synthetic hydroxyapatite (HAp) coatings proved superior effectiveness in stabilizing bone growth around the implant [39,40]. HAp, Ca_10_(PO_4_)_6_(OH)_2_, represents a well-known biocompatible and bioactive material used in restorative and regenerative strategies for hard tissue therapy [41,42,43]. Thanks to its physicochemical and microstructural resemblance to the inorganic phase of natural bone [44], HAp is a preferred replacement for non-immunogenic and bioavailable-limited autografts [45,46], but also for immunogenic and bioavailable xenografts [47,48,49,50]. 

In addition, the osseointegration of bioinert and bioactive materials can be further boosted by coupling or incorporating different growth factors, which encourage repair mechanisms and functional rebuilding of damaged tissue following fractures and other skeletal injuries [51]. Fibroblast growth factors (FGFs) are a group of proteins [52] that regulate the proliferation and differentiation of various cell types, including osteoblasts [53,54]. FGF2 increases osteoblast proliferation and modulates osteoblast differentiation, representing one of the most promising options for hard tissue engineering applications.

A bone implant’s success is strongly related to surrounding tissues’ behavior and their response to the implant’s material. Further, the patient’s health status is of major significance, as high-risk patients are more prone to implant-related complications and implant failure [55,56,57]. Moreover, when tissues around the implanted device are injured, implants’ microbial contamination and implant-associated infections can occur. All implants are susceptible, to some degree, to microbial contamination and colonization phenomena. Implant-associated infections are complications that have a major impact on patient’s life quality and healthcare systems [58]. 

The advent of drug-resistant pathogens and their capacity to produce virulence factors contributed to the pathogenicity and severity of infections related to orthopedic and orthodontic implants [59,60,61]. The presence of bacterial biofilms (well-organized communities of surface-attached bacteria enclosed into a polymeric matrix) defends embedded microorganisms from antibiotics action and immune cells surveillance [62,63]. Their resistance to antibiotics in an adherent state and the probability of their perseverance in tissues, regardless of the implant removal, make the treatment of infection of vital importance.

The avidity with which pathogens colonize implants depends, to some extent, on the implant’s material. Some of the material features are strongly related to bacterial contamination and colonization, such as microstructure and topography, surface chemistry, and chemical composition [64,65].

One effective strategy for preventing opportunistic microbial contamination, circumstantial colonization, and biofilm development on implants is the surface modification with structures that act as local biostatic or biocide systems. In addition to the potentiated osseointegration of implants, HAp-based coatings can also act as ready depots for adsorption and local release of antibiotics [66], such as Ciprofloxacin and Tetracycline [67], Gentamicin [68] and Streptomycin [69]. Consequently, the local release of antimicrobial substances from HAp coatings initiates short-term prophylaxis that reduces microbial adhesion and avoids post-surgical infections currently associated with implant failure.

Coating deposition techniques, such as plasma spraying, chemical vapor deposition (CVD), fusion coating, physical vapor deposition (PVD), thermal or diffusion conversion, radiofrequency magnetron sputtering, sol-gel, and laser-assisted processing methods, have been utilized for the production of HAp coatings [70,71,72]. From these techniques, the matrix-assisted pulsed laser evaporation (MAPLE) method exhibits many advantages, including the possibility to obtain homogeneous coatings with controlled thickness from a wide variety of organic molecules, such as polymers, bioactive substances, or biomolecules [73,74]. 

By gathering the osteoconductive ability of HAp biomaterials and the osteoinductive potential of specific GFs, promising candidates for bone tissue engineering were reported, including scaffolds [75,76], hydrogels [77] and complex constructs [78]. The immobilization/embedding of GFs onto/within coatings does not represent a new strategy to modulate orthopedic or orthodontic implants’ osseointegration. As they are very susceptible to degradation, a relatively soft approach is preferred in order to modify the surface of implants with GFs, mainly by means of physical immobilization [79,80] or non-covalent interactions-guided adsorption [81,82,83,84]. Even if MAPLE is an attractive and versatile technique to transfer biologically active molecules, there is a lack of data regarding its implications on obtaining GF-loaded coatings.

Therefore, in the current study, we aimed to obtain composite coatings based on hydroxyapatite, aminoglycoside antibiotic (Kanamycin), and fibroblast growth factor by MAPLE technique in order to increase the biocompatibility of commercial implant materials by promoting the cell attachment and growth without toxic effects, and the inhibition of microbial biofilm formation.

## 2. Results and Discussions

### 2.1. Physicochemical Investigation of HAp Powder

Chemical co-precipitation represents a facile method to synthesize tunable HAp-based biomaterials, starting from calcium and phosphorous precursors and conveniently adjusting various reaction parameters (temperature range, pH value, nature, and type of templating agent) [85,86]. Moreover, this versatile approach is suitable for extending the bioactivity and biofunctionality of HAp by doping with various inorganic ions [87,88] and by obtaining apatite composites [89,90]. 

**Figure 1:**
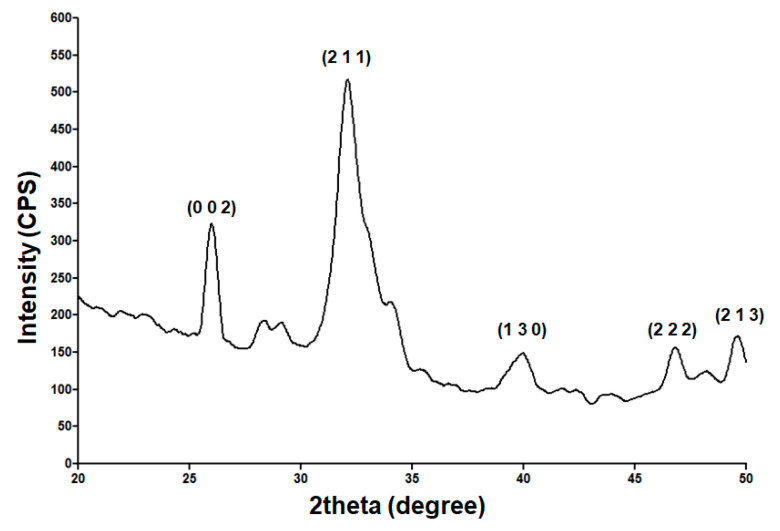
XRD pattern of hydroxyapatite (HAp) powder.

In our case, the white powdery sample resulted after drying the viscous precipitate obtained by chemical synthesis was subjected to compositional and microstructural analysis using the XRD method. The corresponding diffractogram is included in Figure 1. It evidences the presence of broad diffraction peaks, which indicate the powder’s reduced crystallinity. Specific peaks are identified at 2θ values of 25.9°, 28.1°, 28.9°, 31.8°, 34°, 39.8°, 46.7°, and 49.5°. In compliance with PDF Card-01-071-5048 and reported literature [91,92], these maxima correspond to (0 0 2), (1 0 2), (2 1 0), (2 1 1), (2 0 2), (1 3 0), (2 2 2), and (2 1 3) diffraction planes of hydroxyapatite crystals with hexagonal lattice. The XRD analysis confirms that hexagonal HAp represents the sole crystalline phase of the obtained powder.

**Figure 2:**
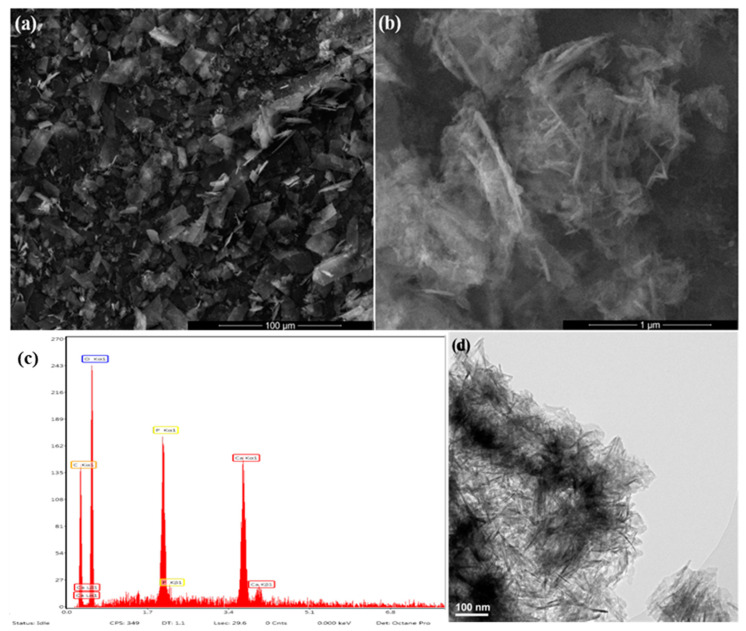
SEM images (**a**,**b**), energy-dispersive X-ray spectroscopy (EDS) spectrum (**c**) and TEM micrograph (**d**) of HAp powder.

From SEM images in Figure 2a,b can be noticed that the HAp powdery sample consists of sharp polyhedral aggregates constituted by individual needle-shaped nanosized particles (width and length of tens and hundreds of nanometers, respectively). Similar morphologies were reported for synthetic hydroxyapatite compared to natural-derived HAp with preferential granular morphology [93,94]. The energy-dispersive X-ray spectroscopy (EDS) spectrum (Figure 2c) confirms the presence of typical elements for HAp: Ca (~3.8 and ~4 keV), P (~2 keV), and O (~0.4 keV). The TEM micrograph from Figure 2d provides intimate microstructural aspects of the HAp powder. One can observe that the previously identified inorganic structures consist in aggregates of individual nanoparticles with preferential plate and rod morphologies (length below 100 nm and width comprised between 5 and 25 nm). This outcome is beneficial for hard tissue engineering applications since the inorganic phase of human bone consists of carbonated apatite nanocrystals with platelet shape (thickness of 1–2 nm, width of 10–80 nm, and length of 15–200 nm) [95,96]. The formation of hexagonal crystalline HAp was confirmed by the corresponding selected area electron diffraction (SAED) pattern (data are not shown).

### 2.2. Physicochemical Investigation of HAp-Based Coatings

**Figure 3:**
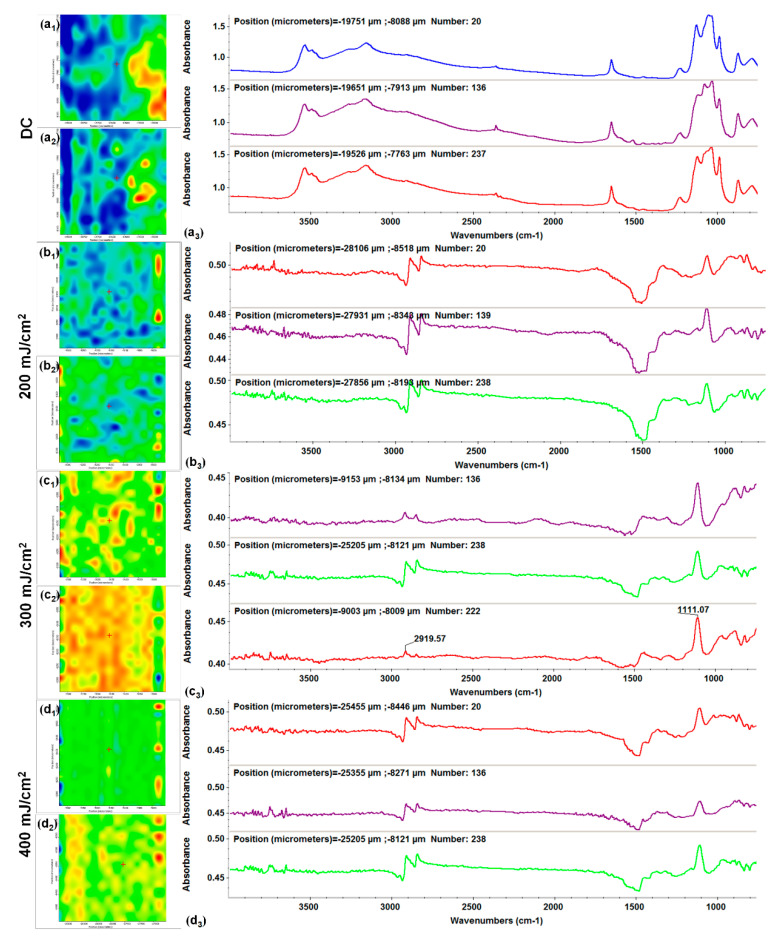
IR maps assigned to the distribution of methylene (**a_1_**,**b_1_**,**c_1,_d_1_**) and phosphate (**a_2_**,**b_2_**,**c_2_**,**d_2_**) groups and IR spectra of dropcast (**a_3_**) and HAp/KAN (Kanamycin) coatings obtained at 200 (**b_3_**), 300 (**c_3_**) and 400 (**d_3_**) mJ/cm^2^ laser fluences.

**Figure 4:**
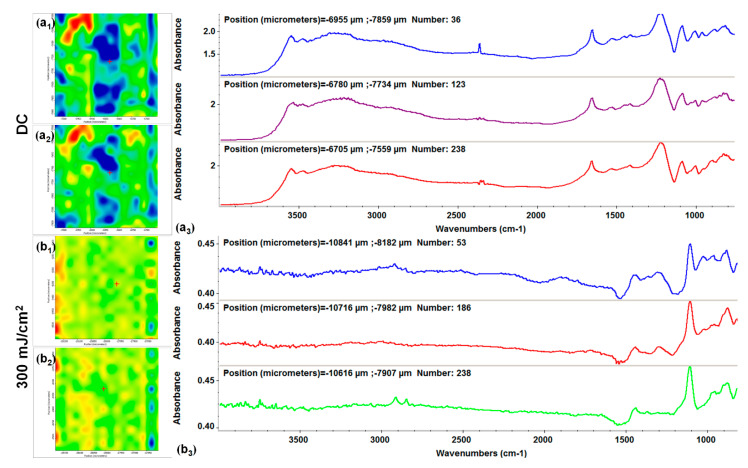
IR maps assigned to the distribution of phosphate (**a_1_**,**b_1_**) and methylene (**a_2_**,**b_2_**) groups and IR spectra of dropcast (**a_3_**) and HAp/KAN/FGF2 (fibroblast growth factor 2) coatings obtained at 300 mJ/cm^2^ laser fluence (**b_3_**)**.**

Being a laser processing technique, which implies high energy levels for material transfer, compositional and microstructural studies are often required to experimentally identify the optimal laser parameters for MAPLE transfer of both inorganic and organic materials, in terms in functional groups integrity and preserved stoichiometry [97,98]. In our case, comparative IR studies were performed on dropcast samples (corresponding to initial materials) and MAPLE coatings obtained at different laser fluences. Complementary infrared maps (with color variations directly related to absorbance intensity) and infrared spectra (with values corresponding to different points on specimens) were recorded for all experimental samples. The IR mapping of all HAp-based materials was performed by monitoring characteristic stretching vibrations of phosphate function (~1100 cm^−1^) within HAp and specific asymmetric and symmetric stretching vibrations of methylene group (2920–2850 cm^−1^ wavenumber range) originating from organic compounds. We decided to perform sequential IR analysis on Kanamycin-embedded HAp coatings (HAp/KAN, Figure 3) and subsequent FGF2-loaded HAp coatings (HAp/KAN/FGF2, Figure 4).

In the IR spectra of initial HAp/KAN material (Figure 3a_3_), absorption maxima that originate from hydroxyapatite can be identified, such as stretching vibrations from structural hydroxyl groups (~3530 cm^−1^), ν3 asymmetric stretching of PO_4_^3−^ (~1110, ~1080, and ~1050 cm^−1^) and PO_4_^3−^ ν1 stretching (~990 and ~880 cm^−1^) [99,100,101]. The weaker peak at ~3500 cm^−1^ may result from the overlapped stretch of OH^−^ (hydroxyl groups bounded in HAp and contained in the carboxylic units of KAN) and N–H (from IR doublet of KAN’s primary amines) [102,103]. C–H group’s small-scaled vibrations, assigned to asymmetric and symmetric vibrations of –CH_2_ from the antibiotic, are noticed at ~2920 and ~2850 cm^−1^. Other IR maxima that confirm the presence of Kanamycin are identified at the following wavenumbers: ~1760 cm^−1^ (strong stretch of carbonyl moiety), ~1520 cm^−1^ (overlapped stretch of –COO^−^ and bend of N–H moieties), and ~1450 cm^−1^ (overlapped stretching vibrations of C=C and C–N) [104,105,106]. The phosphate maxima from 1100 to 1000 cm^−1^ wavenumber may also cover the C-O stretching and HSO_4_^−^ vibrations originating from the antibiotic. 

When compared to the initial composite, the lowest laser fluence (200 mJ/cm^2^) did not affect the chemical integrity of HAp/KAN material (Figure 3b_3_). Slightly modified and reduced IR maxima indicate an insufficient amount of transferred material, as confirmed by the presence of predominant blue areas in the complimentary infrared maps (Figure 3b_1_,b_2_). An increased transfer of HAp/KAN is noticed for the 400 mJ/cm^2^ fluence (Figure 3d_1_,d_2_). However, in terms of efficient and uniform transfer of HAp/KAN material (Figure 3c_1_,c_2_) and preserved chemical integrity (Figure 3c_3_), optimal results are evidenced by using the middle laser fluence (300 mJ/cm^2^).

We identified the 300 mJ/cm^2^ laser fluence as the optimal choice for the MAPLE transfer of uniform and stoichiometric binary coatings from previously discussed infrared data. Therefore, we decided to use only this value to obtain ternary HAp/KAN/FGF2 coatings. The convenient use of this particular laser fluence for the successful and efficient transfer of HAp/KAN/FGF2 composite materials is complementary supported by the IR mapping (Figure 4b_1_,b_2_) and corresponding IR spectra (Figure 4b_3_).

The absorbance maxima previously identified for synthetic apatite can also be noticed in the IR spectra of initial and MAPLE processed HAp/KAN/FGF2 materials (Figure 4a_3_,b_3_, respectively). Alongside, the infrared peaks identified in Figure 4b_3_ at ~3500 cm^−1^ (which may result from overlapped stretching of hydroxyl from HAp and N–H from primary amines of organic compounds), ~2950 and ~2850 cm^−1^ (–CH_2_ vibrations originating from KAN) and ~1460 cm^−1^ (stretching and bending vibrations of carbonaceous bonds from organic molecules), confirm the successful transfer of HAp/KAN/FGF2 composite materials.

Taking into account the above discussed IR data, all composite coatings considered for subsequent SEM analysis, biological and microbiological evaluation were obtained by using the 300 mJ/cm^2^ laser fluence during MAPLE processing. 

**Figure 5:**
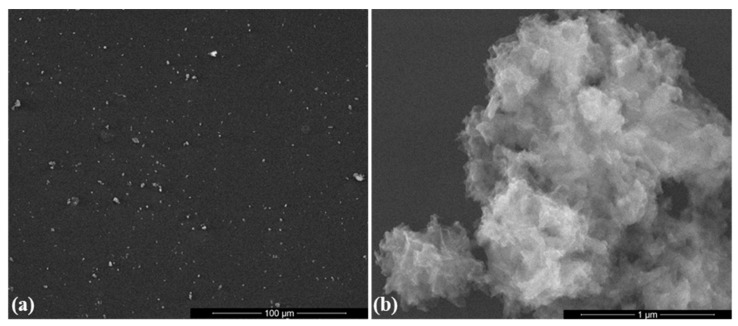
SEM images of HAp/KAN/FGF2 coatings obtained at 300 mJ/cm^2^ laser fluence: (**a**) low magnification and (**b**) high magnification.

As it can be seen in the SEM micrograph from Figure 5a, the selected laser fluence enabled the unaltered and uniform distribution of small aggregates of HAp/KAN/FGF2 composite material onto the substrate. The presence of the sole antibiotic and growth factor did not alter the needle-like morphology of initial HAp particles (data not shown). In the case of HAp/KAN/FGF2 coatings, a distinctive rod-shaped morphology of particles can be noticed, but also the presence of a wavy outer layer (derived from used organic molecules) onto the surface of apatite nanoparticles (Figure 5b). 

### 2.3. Biological Evaluation of HAp-Based Coatings

The biological behavior of medical graded titanium discs modified with HAp-based coatings by MAPLE (performed at 300 mJ/cm^2^ laser fluence) was evaluated on MC3T3-E1 pre-osteoblast cells by using quantitative (MTT viability and NO cytotoxicity assays) and qualitative (fluorescence microscopy) tests. 

In terms of prolonged and superior performance, the beneficial implication of FGF2-containing biomaterials was reported in many studies [107,108]. For example, anodized titanium implants coated with FGF-loaded poly(lactide-co-glycolide) nanoparticles exhibited osteoinductive activity and significantly enhanced the integration of metallic implants [109]. Further, multi-layered polylactide nanosheets loaded with FGF2 significantly accelerated the regeneration process of severe femoral shaft fractures [110]. Moreover, composite coatings of FGF2–apatite led to an augmented interface strength between bone tissue and metallic device in external fixation titanium pins [111].

**Figure 6:**
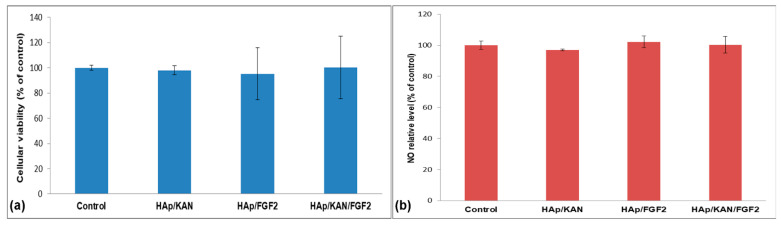
MTT cellular viability (**a**) and NO release (**b**) of osteoblastic cells cultured for 24 h on control and Ti modified with HAp/KAN, HAp/FGF2, and HAp/KAN/FGF2 coatings (*n* = 3)**.**

In our case, the MTT assay (Figure 6a) evidences that all proposed composite coatings are suitable substrates to support the normal growth and proliferation of osteoblastic cells. The metabolic activity of all HAp-coated samples was comparable with that of control (uncoated Ti discs), with viability variations below 10%. Furthermore, Figure 6b shows that the cellular growth on these surfaces did not induce NO release; its level being maintained close to control values for all tested samples (NO release level of 97%, 102%, and 100% for HAp/KAN, HAp/FGF2 and HAp/KAN/FGF2, respectively). Those results correlate very well with previously discussed MTT results. 

**Figure 7:**
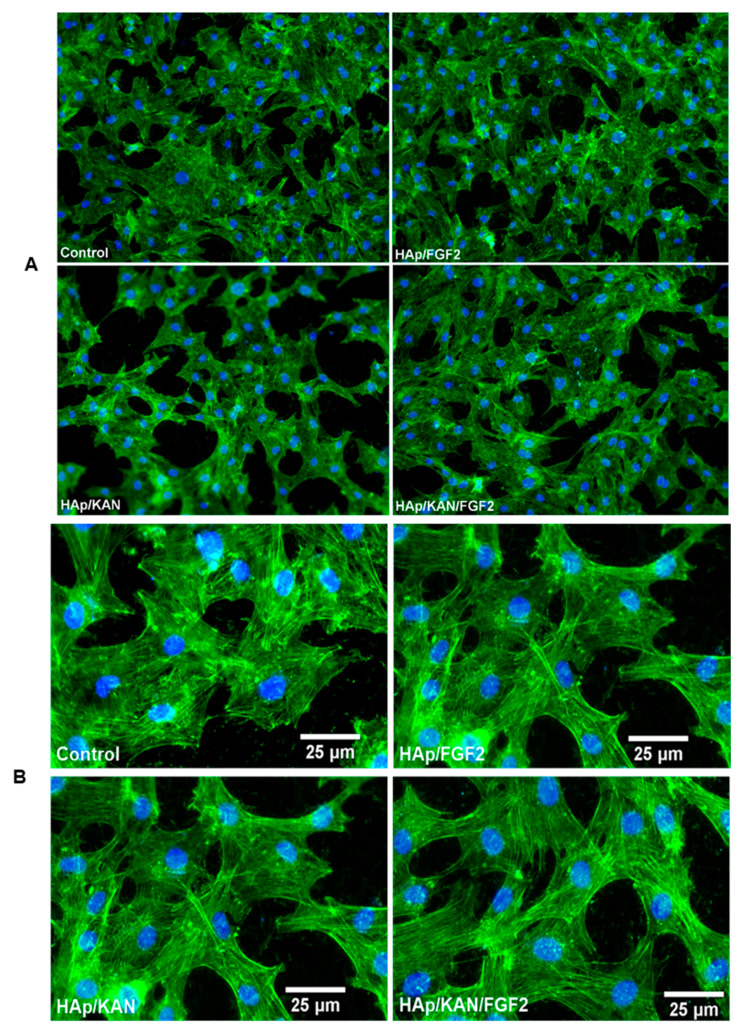
Fluorescence micrographs of osteoblastic cells cultured for 24 h on control and Ti modified with HAp/KAN, HAp/FGF2 and HAp/KAN/FGF2 coatings: (**A**) low magnification with 20× objective and (**B**) high magnification.

The fluorescence micrographs from Figure 7 shows that MC3T3-E1 cells incubated in the presence of HAp-based coatings for 24 h exhibited good adhesion and uniform spreading onto the substrate. The cells also displayed normal morphology and characteristic osteoblast-like phenotype (flattened structure, elongated actin filaments, multiple cytoskeleton extensions, and prominent central nuclei).

### 2.4. Microbiological Evaluation of HAp-Based Coatings

In addition to their intrinsic bioactivity, HAp-based coatings possess an impressive potential for the immobilization or/and encapsulation of antimicrobial substances. Enhanced anti-pathogenic effects were reported for HAp coatings that incorporate inorganic structures, such as bismuth [112], cerium [113], copper [114], magnesium [115] ions, silver ions and nanoparticles [116,117], zinc ions and nanoparticles [118,119]. Nano-textured Ti surfaces coated with calcium phosphate and functionalized with antimicrobial peptides exhibited anti-biofilm and anti-fouling potential against *Escherichia coli* and *Streptococcus mutans* bacteria [120]. HAp-based coatings loaded with Gentamicin showed significant inhibitory effects against *Staphylococcus aureus* [121,122] and *Escherichia coli* [123]. Composite coatings of HAp and poly(lactide-co-glycolide) loaded with Ceftriaxone and Cefuroxime antibiotics significantly impaired Ti substrates’ contamination and colonization with *Escherichia coli* [124].

**Figure 8:**
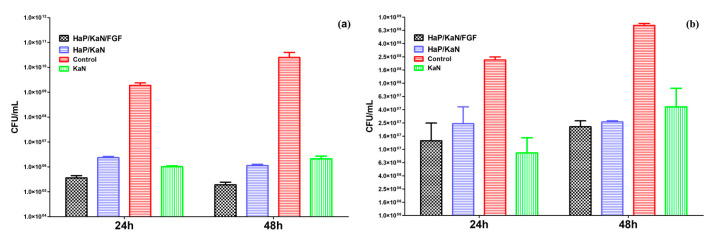
Development of monospecific biofilm of *S. aureus* (**a**) and *Ps. aeruginosa* (**b**) after 24 and 48 h of incubation on control and Ti modified with HAp/KAN and HAp/KAN/FGF2 coatings.

KAN-embedded coatings’ ability to interfere with monospecific bacterial biofilms’ formation and development was assessed against opportunistic strains of *S. aureus* and *Ps. aeruginosa* (Figure 8). Significant inhibitory effects are evidenced against both pathogens. However, a more prominent action is noticed against the Gram-positive strain (Figure 8a). When compared to control samples, the *S. aureus* biofilm development is reduced with more than 3 (24 h), respectively 5 (48 h) orders of magnitude (logs). In *Ps. aeruginosa* strain, the colony-forming units are reduced with maximum 1.5 logs, regardless of the testing time (Figure 8b). Interestingly, the inhibitory effects exhibited by HAp/KAN and HAp/KAN/FGF2 coatings are comparable and more pronounced at 48 h in both situations. These results demonstrate that the proposed composite coatings are efficient against initial contamination and biofilm formation, as well as on the maturation phase of microbial biofilms. Moreover, given that the antimicrobial effect is maintained and even enhanced after 48 h suggests that the obtained materials are stable and preserve their biological properties for at least two days. This period of time is very important when investigating biofilms since after less than 24 h in optimal conditions, biofilms are already mature, and dispersion starts. Maintaining an excellent antimicrobial activity for at least 48 h could also limit the dispersal of biofilms.

Nanostructured coatings present numerous advantages as compared to classical anti-biofilm approaches. The most important traits refer to the fact that nanomodified surfaces could offer a prolonged and controlled release of the antimicrobial agent [125], are able to inhibit initial microbial colonization [126] but also biofilm maturation, and offer a long-lasting effect [127].

The obtained HAp-based nanocoatings proved prolonged biological activity, great biocompatibility, and anti-biofilm efficiency, which are maintained for at least two days. These properties recommend considering the proposed composites as efficient biomedical materials to be used in various applications, such as hard tissue engineering, bone and joint implants, dental medicine, and biocompatible and antimicrobial diagnosis devices/coatings.

## 3. Materials and Methods

### 3.1. Materials

Sigma-Aldrich (Merck Group, Darmstadt, Germany) was the main provider of all reagents used for the synthesis of composite coatings, such as calcium chloride (CaCl_2_), disodium phosphate (Na_2_HPO_4_·2H_2_O), sodium hydroxide (NaOH), and dimethyl sulfoxide (DMSO). Kanamycin sulfate (KAN) and fibroblast growth factor (FGF2) were purchased from the same source. 

IR transparent silicon (Si) substrates (1 cm^2^ area), microscope glass slides (1 cm^2^ area) and commercial graded 2 titanium discs were provided by a local supplier.

The reagents used for cellular assays, namely Dulbecco’s Modified Eagle’s Medium (DMEM), Luria-Bertani (LB) medium, fetal bovine serum (FBS), phosphate buffer saline (PBS), antibiotic mixture, paraformaldehyde (PFA), Triton-X, bovine serum albumin (BSA), isopropanol, tetrazolium salt 3-(4,5-dimethylthiazol-2-yl)-2,5-diphenyltetrazolium bromide (MTT), Griess reagent, green-labeled phalloidin-fluorescein isothiocyanate (FITC) dye and 4′,6-diamidino-2-phenylindole (DAPI) stain, were also purchased from Sigma-Aldrich.

MC3T3-E1 mouse-derived osteoblastic cells (ATCC^®^ CRL-2593), but also *Staphylococcus aureus* (*S. aureus*, ATCC^®^ 25923) and *Pseudomonas aeruginosa* (*Ps. aeruginosa*, ATCC® 27853) strains, were purchased from American Type Culture Collection (ATCC).

### 3.2. Synthesis Methods

#### 3.2.1. Hydroxyapatite (HAp) Synthesis

A co-precipitation protocol was used to synthesize the HAp powdery sample. Calcium-containing and phosphorous-containing aqueous solutions were obtained by dissolving CaCl_2_ and Na_2_HPO_4_·2H_2_O in ultrapure water, respectively. The P-containing solution was dropwise added to the Ca-containing solution, under continuous stirring, followed by alkaline pH adjustment (by NaOH addition). The maturation process (12 h) occurred overnight. The resulted milky solution was subjected to filtration, triple washing treatment, and drying process.

#### 3.2.2. HAp-Based Coatings Synthesis

Before surface modification by MAPLE processing, all substrates were subjected to a triple cleaning treatment in the ultrasonic bath with acetone, ethanol, and deionized water. The HAp-based materials were transferred on double side polished (1 0 0) Si substrates for IR studies, and on glass substrates and titanium discs (12 mm diameter and 0.1 mm thickness) for cellular assays, respectively.

A KrF* excimer laser source (*λ* = 248 nm, *τ*_FWHM_ = 25 ns), model COMPexPro 205 Lambda Physics from Coherent was employed for MAPLE experiments. For solid target preparation, suspensions of HAp/KAN, HAp/FGF2, and HAp/KAN/FGF2 in DMSO (2% concentration) were frozen at liquid nitrogen temperature. During laser processing, several parameters were maintained constant, including substrate temperature and background pressure (room temperature and 0.1 Pa, respectively), target to substrate distance (5 cm), target rotation and laser repetition frequency (0.4 and 20 Hz, respectively), number of applied laser pulses (90,000). The MAPLE coatings were obtained by irradiating the frozen targets at different laser fluences, namely 200, 300, and 400 mJ/cm^2^. 

### 3.3. Physicochemical Investigation

#### 3.3.1. X-ray Diffraction (XRD)

The compositional identification and crystalline structure of the white powdery sample were performed using an XRD-6000 diffractometer from Shimadzu (Duisburg, Germany). The analysis was performed using the Cu_Kα_ radiation (*λ* = 1.056 Å) of the equipment, and the data were collected in the 20–50° range of 2θ diffraction angle. 

#### 3.3.2. Transmission Electron Microscopy (TEM)

The TEM analysis of HAp powder was made with a Tecnai^TM^ G2 F30 S-TWIN high-resolution transmission electron microscope equipped with a selected area electron diffraction (SAED) accessory, from FEI (Thermo Fischer Scientific, Waltham, MA, United States). The instrument operated in the transmission mode (300 kV voltage), with point and line resolutions of 2 Å and 1 Å, respectively. 

#### 3.3.3. Infrared Microscopy (IRM)

The compositional analysis of HAp-based powder and coatings was performed using a Nicolet iN10 MX Fourier transform (FT)-IR microscope from Thermo Fischer Scientific. All scans were recorded in the 4000–600 cm^−1^ wavenumber range (4 cm^−1^ resolution), in the reflection mode. The IR data were processed by using the OmincPicta 8.2 software (Thermo Fischer Scientific).

#### 3.3.4. Scanning Electron Microscopy (SEM)

SEM investigation was performed on pristine HAp powder, as well as on composite coatings obtained by MAPLE. Before analysis, all samples were capped with a thin conductive gold layer. The micrographs were recorded using the secondary electron beam (30 keV) of an electronic microscope equipped with energy-dispersive X-ray spectroscopy (EDS) accessory from FEI (Thermo Fischer Scientific).

### 3.4. Biocompatibility Evaluation

Complementary data on the biological behavior of HAp/KAN, HAp/FGF2 and HAp/KAN/FGF2 coatings were obtained in the presence of MC3T3-E1 murine osteoblast cultures. Before both quantitative and qualitative assays, the cells were cultured in DMEM supplemented with 10% FBS and antibiotic mixture, at 37 °C, in a humid atmosphere with 5% CO_2_. All specimens, namely uncoated substrates (control) and substrates modified by MAPLE processing, were sterilized by UV exposure for one hour before cellular assessment.

#### 3.4.1. MTT Cell Viability Assay

To quantitatively evaluate viable cells, the MTT colorimetric test was used. This method relies on the enzymatic reduction of the tetrazolium salt to its insoluble formazan, which only occurs in metabolically active cells. MC3T3-E1 cells were seeded in the presence of uncoated and MAPLE-coated substrates at 2 × 10^4^ cells/cm^2^ cellular density. The cellular density and incubation time used within this work were in agreement with similar studies previously performed, being the proper choices for this kind of biocompatibility tests [128,129,130].

The culture medium was removed after 24 h of standard incubation. It was replaced with MTT solution (1 mg/mL) and followed by 4 h of incubation in dark conditions. The water-insoluble formazan crystals were dissolved with isopropanol, and the optical density of the resulted medium (directly related to the number of metabolically active cells) was spectrophotometrically measured at 595 nm, using a FlexStation 3 multi-mode microplate reader from Molecular Devices (California, United States).

#### 3.4.2. Nitric Oxide (NO) Cell Cytotoxicity Assay

An adapted protocol was used to quantify nitric oxide (NO) concentration within the culture medium previously collected after 24 h of incubation. This assay is based on the colorimetric detection of an azo dye, which results by mixing culture supernatants with Griess reagent, a stoichiometric solution (*v/v*) of 0.1% naphthylethylendiamine dihydrochloride and 1% sulphanilamide in 5% H_3_PO_4_. Being directly connected with inflammation and apoptosis processes, increased levels of NO are related with cytotoxic effects. The absorbance of as-obtained solutions was read at 550 nm using the FlexStation 3 multi-mode microplate reader. The NO concentration was calculated from the NaNO_2_ standard curve.

#### 3.4.3. Fluorescence Microscopy

Additional information on composite coatings’ biological behavior was provided by fluorescence microscopy studies, which enable qualitative cellular analysis by simultaneous visualization of the cytoskeleton (due to phalloidin’s stabilizing action to F-actin filaments) and nuclei (due to increased affinity of DAPI stain for DNA structure). After standard incubation for 24 h in the presence of control and coated samples, the culture medium was removed. The cells were fixed for 20 min with 500 µL of 4% PFA solution (in PBS) and permeabilized for one hour with 500 µL of 0.1% Triton-X/1.2% BSA (in PBS). Following the additional dark incubation of as-treated cells with phalloidin-FITC dye (20 μg/mL) and DAPI (2 μg/mL), cells were triply washed with PBS and visualized under an Olympus IX71 fluorescence microscope (Tokyo, Japan).

### 3.5. Microbiological Evaluation

One Gram-positive (*S. aureus* ATCC^®^ 25923) and one Gram-negative (*Ps. aeruginosa* ATCC^®^ 27853) microbial model species with biomedical impact were used to assess KAN-embedded composite coatings’ ability to interfere with microbial colonization and development of bacterial biofilms. Prior to cellular tests, all samples (uncoated and MAPLE-coated specimens) were sterilized by UV exposure for 20 min.

The as-treated specimens were individually placed in sterile 6 well plates (Nunc) with 2 mL of LB broth and inoculated with 20 μL of bacterial suspensions of 0.5 McFarland standard densities (corresponding to 1.5 × 10^8^ CFU/mL). Following their incubation under standard conditions for 24 and 48 h, the specimens were washed with sterile phosphate-buffered saline (PBS), then transferred in sterile tubes containing 1 mL of fresh PBS, which were vigorously vortexed for 30 s. The as-resulted bacterial suspensions (containing biofilm-forming cells) were subjected to ten-fold serial dilution, and 10 µL of each dilution was seeded in triplicate onto LB agar plates. After 20 h of additional incubation, the viable cell count assay was performed to estimate the colony-forming units (CFU/mL) values. The experiments were performed in triplicate and repeated on three separate occasions.

### 3.6. Statistical Analysis of Data

Biocompatibility and microbiological results were performed in triplicate (*n* = 3) and analyzed using GraphPadIn Stat and Prism software by applying One-way Analysis of Variance (ANOVA) test. Statistically significant data were considered for *p* < 0.05.

## 4. Conclusions

This study reports on a multifunctional composite coating based on hydroxyapatite and bioactive molecules, such as antibiotics and growth factors. HAp/KAN/FGF2 coatings successfully inhibited biofilm formation in vitro with no harmful effects on murine cells, but with an excellent cell adherence and spreading on the as-modified surfaces. The MAPLE processed thin coatings proved to be good candidates for the design of efficient implants and surfaces, with a significant impact in hard tissue engineering applications.

## Data Availability

The data presented in this study are available on request from the corresponding author.
